# Miscellany

**Published:** 1903-04

**Authors:** 


### Miscellany.

A FOSSIL UNEARTHED IN AUSTRIA.—Some two years ago, while tending some roots of the vines in a vineyard at Attenburg, Lower Austria, a gardener unearthed the lower jaws and upper molars of a gigantic animal, presumably a rhinoceros, which were taken to the high school at Vienna for further investigation. Professor Toula closely examined the relics, and recognized from the structure of the teeth that the remains were not those of the ordinary woolly rhinoceros. He immediately repaired to the vineyard, where he continued excavations at the point where the skull was disinterred, and discovered practically the whole of the skeleton of this interesting animal, which has now been mounted. Although a portion of the skull is missing, there is sufficient to show that the beast was of the two-horned species found in Sumatra. The breccia where the skeleton was found is of the Pleistocene age. It also contained the remains of a goat.—*Scientific American.*

---

An Oxyacetylene Blow-Pipe.—An oxyacetylene blow-pipe is described by M. Fouche in the Bulletin of the French Physical Society. The flame is formed by the combustion of a mixture of one part of acetylene to one-eighth of oxygen, and in order that the explosion may not travel back into the blow-pipe, a jet velocity is required due to the pressure of a water column four metres in height. The flame melts most metals readily; it will solder iron and steel. Even silica and lime are melted by it. With a reduction of the proportion of oxygen, the flame becomes luminous, and on falling on lime the free carbon goes to form carbide of lime.—*Scientific American.*

---

To perfectly fit Clasps.—Dr. Geo. W. Pitts, at the ninth annual clinic of the Chicago College of Dental Surgery Alumni Association, demonstrated a method of perfectly fitting clasps for partial dentures.

Thin platinum is first burnished around the tooth to be clasped and extended slightly into the sulci. The clasp is then fitted over the platinum, the two waxed together, removed, invested, and solder flowed between them and over the platinum projecting between the cusps. The idea of the clinician being that the platinum may be perfectly adapted to the tooth, whereas clasp metal could not be, and that prevention of crowding upon the tissues is prevented by the projections upon the occlusal surface.—*Dental Review.*

---

Chemical Test for Sodium Dioxide.—Place about one gramme of the sodium dioxide powder in a clean, dry test-tube and add one cubic centimetre of water. If the chemical is efficient, enough oxygen will be generated to kindle a splinter to glowing heat when held at the mouth of the tube.—J. P. BUCKLEY, *Dental Review.*

